# Xiao-Qing-Long-Tang Maintains Cardiac Function during Heart Failure with Reduced Ejection Fraction in Salt-Sensitive Rats by Regulating the Imbalance of Cardiac Sympathetic Innervation

**DOI:** 10.1155/2020/9467271

**Published:** 2020-11-24

**Authors:** Zhaoyu Li, Yongcheng Wang, Yuehua Jiang, Dufang Ma, Ping Jiang, Guofeng Zhou, Jinlong Yang, Feng Dong, Hengyi Zhao, Yimei Zhang, Xiao Li

**Affiliations:** ^1^First Clinical Medical College, Shandong University of Traditional Chinese Medicine, Jinan 250014, China; ^2^Department of Cardiovascular, Affiliated Hospital of Shandong University of Traditional Chinese Medicine, Jinan 250011, China; ^3^Central Laboratory, Affiliated Hospital of Shandong University of Traditional Chinese Medicine, Jinan 250011, China; ^4^General Medicine, Affiliated Hospital of Shandong University of Traditional Chinese Medicine, Jinan 250011, China; ^5^Department of Acupuncture and Moxibustion, Second Affiliated Hospital of Shandong University of Traditional Chinese Medicine, Jinan 250001, China

## Abstract

**Objective:**

The anatomical and functional imbalances of sympathetic nerves are associated with cardiovascular disease progression. Xiao-Qing-Long-Tang (XQLT), an ancient Chinese herbal formula, has been used to treat cardiovascular diseases in eastern Asia for thousands of years. We determined the effect of XQLT in maintaining cardiac function during heart failure with reduced ejection fraction (HFrEF) with respect to its neurobiological effects in salt-sensitive rats.

**Methods:**

Dahl salt-sensitive (DS) rats were fed a high-salt diet to establish an HFrEF model and were divided into model (DS, administered normal saline) and XQL groups (administrated XQLT) randomly, with SS-13BN rats being used as the control. The bodyweight and blood pressure of rats were observed regularly. Electrocardiogram, echocardiography, and plasma N-terminal pro-B-type natriuretic peptide (NT-proBNP) were determined to assess cardiac function. The sympathetic tune and myocardial morphological changes were evaluated. Western blot and qRT-PCR were used to assay the expression of the nerve growth factor (NGF) and leukemia inhibitory factor (LIF). Tyrosine hydroxylase (TH), choline acetyltransferase (CHAT), and growth-associated protein 43 (GAP43) were assayed to confirm sympathetic remodeling. The micromorphological changes in cardiac sympathetic nerve endings were observed by transmission electron microscopy.

**Results:**

Four weeks after XQLT treatment, cardiac function and bodyweight were higher and blood pressure was lower than that of the DS group. Myocardial noradrenaline (NA) increased, while the plasma NA level decreased significantly. The morphology demonstrated that XQLT significantly alleviated myocardial damage. XQLT decreased the expression of LIF, increased the expression of NGF, enhanced the TH+/GAP43+ and TH+/CHAT + positive nerve fiber density, and improved the TH and GAP43 protein expression, but had no effect on CHAT. Moreover, XQLT improved the micromorphology of sympathetic nerve endings in the myocardium.

**Conclusion:**

XQLT maintains cardiac function during HFrEF in salt-sensitive rats, in part, by regulating the imbalance of cardiac sympathetic innervation.

## 1. Introduction

Cardiac sympathetic activation is an important biological feature of heart failure (HF) [1]. When HF occurs, the neuroendocrine system compensates, and the sympathetic and parasympathetic activities are unbalanced, which in turn promote cardiac compensatory remodeling and development of heart failure with reduced ejection fraction (HFrEF) [2, 3]. Sympathetic remodeling is considered an important mechanism of HFrEF pathogenesis, which is manifested by the imbalance of anatomy and function, including sympathetic regenerate, and cholinergic transformation. Reversing sympathetic remodeling is of great significance for the treatment of HFrEF. Recent studies show that normal sympathetic innervation helps maintain the structure and function of the heart and plays a key role in regulating myocardial function and in maintaining cardiovascular homeostasis [4, 5]. Our previous studies also found that reversing sympathetic remodeling and maintaining sympathetic-vagal balance reduced the damage of sympathetic nerves and myocardium in diabetic rats [6, 7]. Therefore, the treatment of HFrEF should not emphasize the inhibition of sympathetic nerves and should aim at restoring the sympathetic innervation balance.

Nerve growth factor (NGF) and leukemia inhibitory factor (LIF) play important roles in affecting the anatomy and function of sympathetic nerves in HF. NGF first increases and then decreases during the development of HF. Increased expression of NGF in early HF leads to higher sympathetic nerve density, while decreased expression of NGF in late HF leads to lower sympathetic innervation density [8, 9]. Continuous elevation of LIF during the whole process of HF leads to sympathetic regeneration and cholinergic transdifferentiation, resulting in the functional denervation of the sympathetic nerves [10, 11]. Accordingly, we demonstrated that regulating the imbalance of NGF and LIF may improve the anatomical and functional denervation of sympathetic nerves in HFrEF.

XQLT is one of the classic prescriptions of traditional Chinese medicine and is used for treating exogenous cold and interior fluid. The theory of traditional Chinese medicine holds that yang deficiency, as well as exogenous cold and fluid retention, is related to HF. On this basis, XQLT has been used to treat cardiovascular diseases for thousands of years. Modern clinical researchers have found that XQLT has a therapeutic effect on pulmonary heart disease, chronic heart failure (CHF), as well as other cardiac diseases [12, 13]. Even though the basic in vitro or in vivo studies on XQLT mainly focus on the treatment of lung diseases. XQLT has been found to exhibit anti-inflammatory and antiallergic reactions in an allergic asthma mouse model [14]. Our previous study found that XQLT prevented the development of HF by regulating the composition of the gut microbiota [15], which suggested that XQLT led to beneficial effects in the progression of HF. However, as an effective prescription for HF, the effect of XQLT on the imbalance of cardiac sympathetic innervation is still unclear and whether the effect of XQLT on cardiac sympathetic nerves is involved in improving HFrEF needs to be demonstrated.

In this study, we focused on the imbalance of cardiac sympathetic innervation and aimed to explore the mechanism underlying the cardiac sympathetic innervation in rats with HFrEF. The data suggested that XQLT could maintain cardiac function in salt-sensitive rats with HFrEF by regulating the imbalance of NGF and LIF.

## 2. Materials and Methods

### 2.1. Animals and Grouping

Twenty-seven 6-week-old specific pathogen-free male rats weighing 160–200 g, including eighteen Dahl salt-sensitive (DS) rats and nine SS-13BN rats, were provided by the Beijing Vital River Laboratory Animal Technology Co., Ltd. (Beijing, China) (Permit No. SCXK (JING) 20160011). All animal experiments in this project were approved by the Institutional Animal Care and Use Committee of Shandong University of Traditional Chinese Medicine (No. SDUTCM2018071501). The rats were housed under conditions of ambient temperature (23 ± 2°C) and a 12 : 12 h light-dark cycle, with tap water and food provided ad libitum, for a week before the experiment. After the adaptation period, the animals were distributed into the SS-13BN (control, *n* = 9), DS (model, *n* = 9), and XQL (*n* = 9) groups. All the rats were fed with a high-salt diet (8%, NaCl). After 10 weeks, the HF model was successfully established.

### 2.2. Preparation of XQLT and Intervention

The daily prescription of XQLT for one adult was as follows: *Ephedra sinica* Stapf (6 g), *Ramulus Cinnamomi* (15 g), *Radix Paeoniae Alba* (15 g), *Asarum sieboldii* Miq. (3 g), *Schisandra chinensis* (9 g), *Pinellia ternata* (9 g), and *Radix Glycyrrhizae* (9 g); all were purchased from the affiliated hospital of Shandong University of Traditional Chinese Medicine, and herbs were identified by Prof. Chuanjiang Ma of TCM Pharmacy. The herbs were mixed and extracted twice under reflux with distilled water (1 : 10 volume), 1 h per extraction. The solution was combined and concentrated to a relative density of 1.20–1.25 g/mL (70–80°C). Two granules/g raw herbs were prepared to obtain XQLT granules. The solution density was 1.2 g raw medicinal herbs/mL. After 10 weeks of a high-salt diet, the rats in the XQL group received intragastric administration with XQLT, while those in SS-13BN and DS groups received normal saline. Rats were administered with the substances by gavage once a day for 4 weeks. All the rats were fed the same diet as before during the drug administration.

### 2.3. Mean Arterial Pressure and Bodyweight

Using tail-cuff blood pressure multichannel systems (MRBP, USA), mean arterial pressure (MAP) of rats was measured from 8 : 00 am to 12 : 00 am from Monday to Wednesday every third week [16]. All rats' tails were prewarmed at 30°C for 15 min before measurement. Mean arterial pressure was measured three times repeatedly with an interval of 30 s, and the average was recorded as the blood pressure value. The bodyweights of rats were periodically recorded every week.

### 2.4. Electrocardiogram and Echocardiography

After continuous dosing, the rats were anesthetized by intraperitoneal injection of 4% sodium pentobarbital (20 mg/kg) and then received an electrocardiogram (ECG) [17]. Electrocardiogram was recorded at a paper speed of 25 mm/sec, and the standard voltage was 10 mv/mm. The PR interval and the heart rate were assessed. At the end of 6, 10, and 14 weeks, the rats were subjected to echocardiography after anesthesia to compare the cardiac function at different time intervals, determined using an M5 Vet Veterinary Ultrasound system (Mindray, China) [6]. The indexes included interventricular septal thickness (IVSd) and left ventricular ejection fraction (LVEF).

### 2.5. Blood Sampling and Collection of Left Ventricle Tissue

After treatment, each rat was euthanized by cervical dislocation under anesthesia administered by intraperitoneal injection of 4% sodium pentobarbital (20 mg/kg). Blood samples were collected from inferior vena cava. Serum was separated by centrifuging at 3000 rpm for 5 min at 4°C and stored at −80°C for ELISA assay. The left ventricles were removed as soon as possible on ice and divided into three parts: one part was fixed in 10% neutral formalin for H&E staining and Masson staining. The second part was frozen in liquid nitrogen for ELISA, Western blot, qRT-PCR, and immunofluorescence. Another was fixed for transmission electron microscopy.

### 2.6. H&E and Masson Staining

The left ventricles were fixed in 10% neutral formalin and embedded in paraffin. Histological sections (4 *μ*m thick) were subjected to hematoxylin and eosin (HE, Solarbio, China) and Masson's trichrome (Masson, Solarbio, China) staining. Images were obtained in three random fields per section [18]. The inflammatory cell infiltration was assessed by analysis of the H&E staining, and collagen volume fraction was assessed by analysis of the Masson staining. The analysis was performed with the Image J software (NIH, USA).

### 2.7. Sandwich ELISA

The plasma levels of N-terminal pro-B-type natriuretic peptide (NT-proBNP) were measured using high-sensitivity enzyme-linked immunosorbent assay (ELISA) kits (NT-proBNP, CUSABIO). And norepinephrine (NA) in plasma and LV tissue were determined with ELISA kits: NA (CUSABIO). All tests were performed according to the user manuals.

### 2.8. Real-Time Quantitative PCR (qRT-PCR)

Total RNA was extracted from the left ventricle using TRIzol (Invitrogen, USA), and reverse transcription for cDNA synthesis was performed by a PrimeScript RT reagent kit with gDNA Eraser (Takara, Japan). The reverse transcription reaction was conducted at 42°C for 15 min and 85°C for 5 s. The prepared cDNA was amplified by PCR at 95°C for 30 s, followed by 40 cycles at 95°C for 5 s and 60°C for 30 s. Then, the dissociation procedure was performed at 95°C for 5 s, 60°C for 1 min, and 95°C for 15 s. The sequences of forward/reverse primers (Synthesized by Takara, China) were as follows: NGF: 5′-TGCCAAGGACGCAGCTTTC-3′/5′-TGAAGTTTAGTCCAGTGGGCTTCAG-3′; LIF: 5′-ATCAAGAGTCAACTGGCTCAACTCA-3′/5′-TGTTGGGCGCACATAGCTTATC-3′; and GAPDH: 5′-ATGACCCCTTCATTGACCTCA-3′/5′-GAGATGATGACCCTTTTGGCT-3′. Next, qRT-PCR was conducted using the Light Cycler 480 SYBR Premix Ex Taq II (Roche, Germany). The mRNA expression levels of NGF, LIF, and GAPDH were detected, and GAPDH was used for normalization. The relative gene expression in the sample was calculated as 2^−ΔΔCT^. Experiments were performed in triplicate [19].

### 2.9. Western Blot Analysis

Proteins were extracted from the left ventricle tissue. The tissues were homogenized in ice-cold RIPA buffer, and protein concentrations were determined using the enhanced bicinchoninic acid (BCA) protein assay kit (Beyotime Biotechnology, China). The 30 *μ*g of proteins underwent electrophoretic separation by sodium dodecyl sulfate-polyacrylamide gel electrophoresis (SDS-PAGE) and were transferred to polyvinylidene fluoride (PVDF) membranes for blotting. The membranes were blocked with 5% nonfat milk in TBST for 1 h at room temperature and, subsequently, incubated overnight at 4°C with primary antibodies against NGF (Abcam, Cat No. ab52918, 1 : 1000), LIF (Abcam, Cat No. ab113262, 1 : 1000), TH (Abcam, Cat No. ab137869, 1 : 5000), CHAT (Abcam, Cat No. ab178850, 1 : 1000), growth-associated protein 43 (GAP43) (Abcam, Cat No. ab16053, 1 : 1000), and *β*-actin (Proteintech, Cat No. 20536-1-AP, 1 : 5000). After the membrane was washed five times (5 min each) in TBST, the blots were incubated for 1 h with goat anti-rabbit IgG (ZSGB-Bio, Cat No. ZB2301, 1 : 10000). The intensity of each band was measured using FluorChem Q 3.4 (ProteinSimple, USA).

### 2.10. Immunofluorescence

Frozen samples were fixed in paraformaldehyde and cut into 8 *μ*m sections; then, antigen was recovered and blocked and incubated with the primary antibodies overnight at 4°C. Afterward, we observed reaction products by secondary antibodies, and the sections were counter-stained with DAPI for 10 minutes. The following primary antibodies were used: rabbit anti-rat TH (Abcam, ab112, 1 : 100) was used to stain noradrenergic nerve fibers, and cholinergic cell bodies and nerve fibers were stained using goat anti-rat CHAT (Novus, NBP1-30052, 1 : 100). And mouse anti-rat GAP43 (Abcam, ab129990, 1 : 100) was used to localize sympathetic regeneration in the heart. The secondary antibodies used were as follows: donkey anti-rabbit IgG H&L (Abcam, ab150075, 1 : 200), donkey anti-goat IgG H&L (Abcam, ab6881, 1 : 500), and donkey anti-mouse IgG H&L (Abcam, ab150105, 1 : 600). The stained slides were observed under a Zeiss Vert A1 fluorescence microscope (Carl Zeiss Jena, German) [20]. Images were obtained in ten random fields per section, and analysis was performed with Image-Pro Plus analysis software (Media Cybernetics, USA).

### 2.11. Transmission Electron Microscopy (TEM)

The left ventricles were dissected into 1 mm^3^ pieces and fixed in 2.5% glutaraldehyde and 2% paraformaldehyde/0.1 M cacodylate buffer (pH 7.4) at 4°C. Subsequently, the samples were fixed in 2% osmium tetraoxide for 2 h, dehydrated in graded alcohol, embedded in Epon 812, and stained with uranyl acetate and lead citrate. Transmission electron microscopy was used to observe the changes in the microstructure of sympathetic nerve endings and the changes in small granular vesicles (SGVs, involving catecholamine) and small agranular vesicles (SAGVs, involving Ach) [21]. This project was commissioned by Shandong Weiya Biotechnology Co., Ltd.

### 2.12. Statistical Analysis

Statistical analyses of data were performed by using the SPSS 22.0 software. Quantitative data were presented as mean ± SD and analyzed by single-factor analysis of variance (ANOVA) followed by Dunnett's test or the Student–Newman–Keuls test. *P* < 0.05 was considered as statistical significance.

## 3. Results

### 3.1. XQLT Improved Physical Condition and Cardiac Function

**Figure 1:**
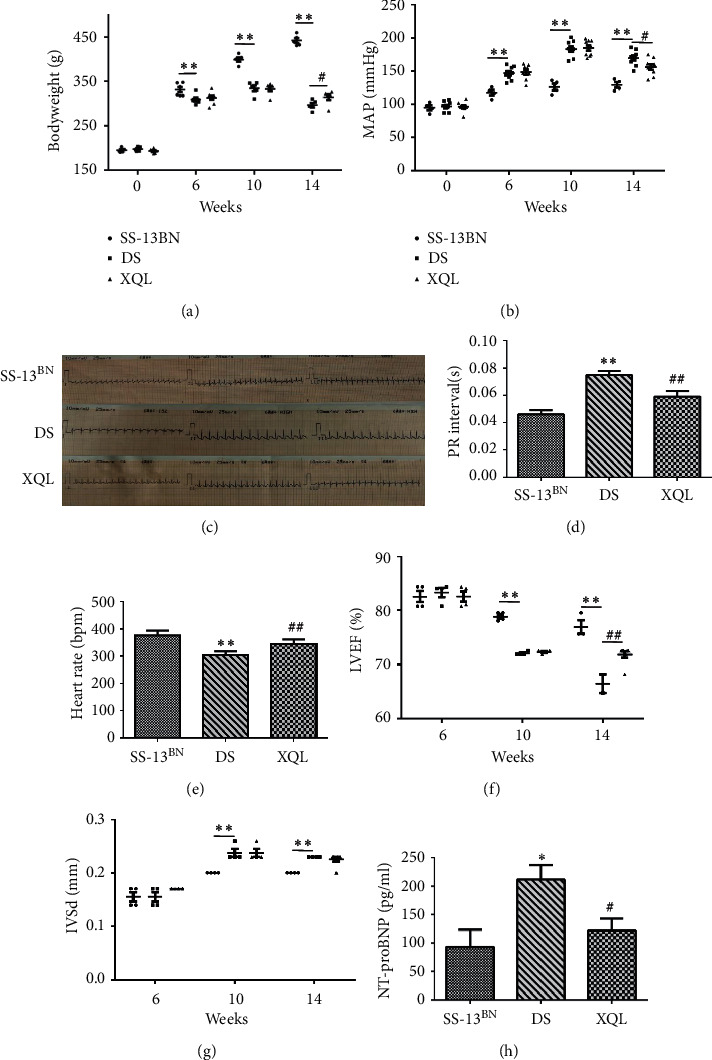
Evaluation of physical condition and cardiac function. (a) Body weight, (b) blood pressure, (c) representative electrocardiographic images, (d) PR interval, (e) heart rate, (f) LVEF, (g) IVSd, and (h) NT-proBNP were measured from different groups. The data were presented as the mean ± SD, *n* = 9. ^*∗*^*P* < 0.05, ^*∗∗*^*P* < 0.01 vs. the SS-13^BN^ group; ^#^*P* < 0.05, ^##^*P* < 0.01 vs. the DS group.

The bodyweight of SS-13BN rats was much heavier than that of DS and XQL rats at the end of the 14^th^ week (*P* < 0.01). Compared to the DS group, the bodyweight of the XQL group showed a significantly slower loss trend during 14 weeks (SS-13BN 442.28 ± 12.20 g, DS 296.46 ± 11.20 g, and XQL 314.15 ± 15.29 g, *P* < 0.05, Figure 1(a)). After 10 weeks of a high-salt diet, the blood pressures of DS and XQL groups reached the highest value, and the XQLT had not yet exerted a significant blood pressure lowering effect. After 14 weeks, XQL significantly lowered the blood pressure of DS rats (SS-13BN 128.92 ± 8.71 mmHg, DS 169.70 ± 11.38 mmHg, and XQL 156.16 ± 12.78 mmHg; *P* < 0.05; Figure 1(b)).

We selected I-III leads to observe the differences in ECGs among the groups (Figure 1(c)). Abnormal changes in heart rate and PR interval are markers of an atrioventricular block [22]. Both DS and XQL groups showed an increase in PR interval (SS-13BN 0.046 ± 0.003 s, DS 0.075 ± 0.003 s, and XQL 0.059 ± 0.004 s; *P* < 0.01; Figure 1(d)) and a decrease in heart rate (SS-13BN 376.1 ± 18.3 bpm, DS 303.3 ± 14.0 bpm, and XQL 345.4 ± 16.1 bpm; *P* < 0.01; Figure 1(e)) compared to those of the SS-13BN group. XQL shortened PR interval and increased heart rate. Furthermore, LVEF of DS and XQL groups was significantly decreased compared with that of the SS-13BN group (*P* < 0.01), and the XQL group showed a significantly slower downward trend than did the DS group (SS-13BN 76.97 ± 2.23%, DS 66.45 ± 2.45%, and XQL 71.83 ± 1.63%; *P* < 0.01; Figure 1(f)). IVSd of DS and XQL groups increased to the highest value on the 10^th^ week. However, XQL demonstrated no effect on IVSd (Figure 1(g)). NT-proBNP is a well-accepted factor in evaluating heart function. Compared to the SS-13BN group, the NT-proBNP of DS and XQL groups was of higher level (*P* < 0.05). Compared with that in the DS group, NT-proBNP was decreased in the XQL group (*P* < 0.05, Figure 1(h)).

### 3.2. XQLT Promoted Histological Recovery

**Figure 2:**
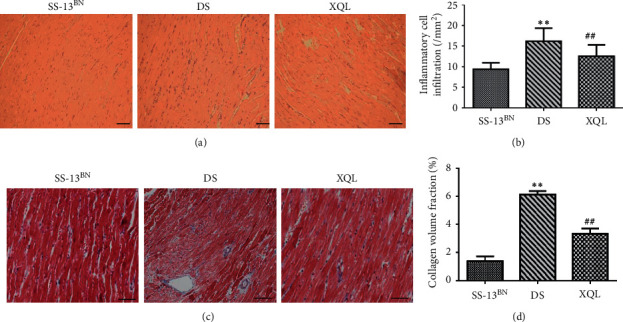
Analysis of myocardial pathology by light microscopy. (a) Representative images of H&E-stained cardiac tissue. (b) Infammatory cell infltration was assessed by analysis of the H&E staining. (c) Representative images of Masson-stained cardiac tissue. (d) Collagen volume fraction of the Masson staining (*n* = 9, scale bars: 50 *μ*m).

The H&E and Masson staining showed the changes in myocardial morphology (Figures 2(a) and 2(c)). Cardiac cells in the SS-13BN group were distributed in order. In the DS group, the myocardial distribution was disordered, the interstitial space was edematous, inflammatory cells were infiltrated, and collagen was deposited. In the XQL group, the myocardial arrangement was more regular, the intercellular space became smaller, the inflammatory cell infiltration, and collagen deposition were reduced compared to those of the DS group.

The inflammatory cell infiltration was assessed by analysis of the H&E staining and was less for the XQL group compared to that of the DS group (Figure 2(b)). Masson staining showed that there was significantly less collagen deposition of the left ventricle in the XQL group than that in the DS group, although the collagen volume fraction of the left ventricle in the XQL group was higher than that in the SS-13BN group (SS-13BN 1.37 ± 0.36%, DS 6.13 ± 0.25%, and XQL 3.34 ± 0.38%; *P* < 0.01; Figure 2(d)).

### 3.3. XQLT Regulated the Expression of NA, NGF, and LIF

**Figure 3:**
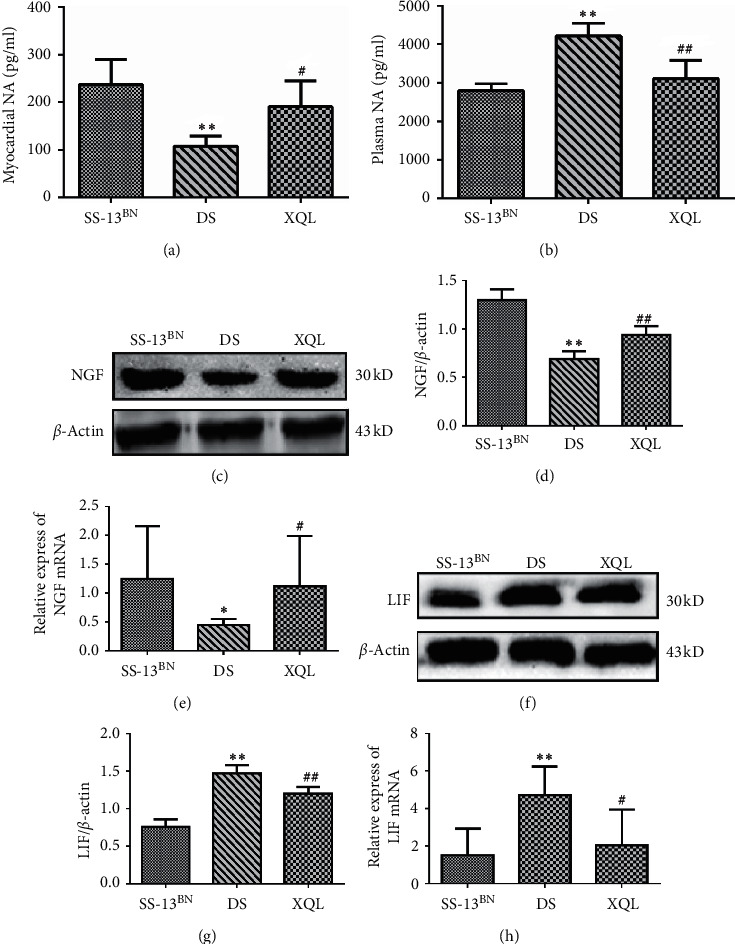
Analysis of the expression of NA, NGF, and LIF. (a), (b) Detection of NA concentrations in left ventricle and plasma by ELISA, respectively. (c) Representative Western blot of myocardial NGF. (d) Relative protein level was calculated by band intensity against *β*-actin. (e) The mRNA expressions of NGF in left ventricle tissues were determined by qRT-PCR. The levels of myocardial LIF were measured by (f) Western blot, (g) band intensity against *β*-actin, and (h) qRT-PCR. Quantitative data are shown as the mean ± SD, *n* = 9, ^*∗*^*P* < 0.05, ^*∗∗*^*P* < 0.01 vs the SS-13^BN^ group; ^#^*P* < 0.05, ^##^*P* < 0.01 vs. the DS group.

Both DS rats and XQL rats had lower levels of NA in myocardial tissues (*P* < 0.01) and higher levels of plasma NA (*P* < 0.01) compared to those of the SS-13BN rats. After 4 weeks of administration, XQLT increased the myocardial NA (*P* < 0.05) and significantly decreased the plasma NA (*P* < 0.01) content as compared to that of the DS group (Figures 3(a) and 3(b)).

As shown in Figures 3(c)–3(h), compared to the SS-13BN group, the expressions of NGF mRNA (*P* < 0.05) and protein decreased (*P* < 0.01), while LIF increased (*P* < 0.01) in the DS group. At 14 weeks, the XQLT increased the NGF mRNA (*P* = 0.01) and protein levels (*P* < 0.01) while simultaneously lowering LIF expression (*P* < 0.05) compared to those of the DS group.

### 3.4. XQLT Improved the Anatomical Dominance of Sympathetic Nerves

**Figure 4:**
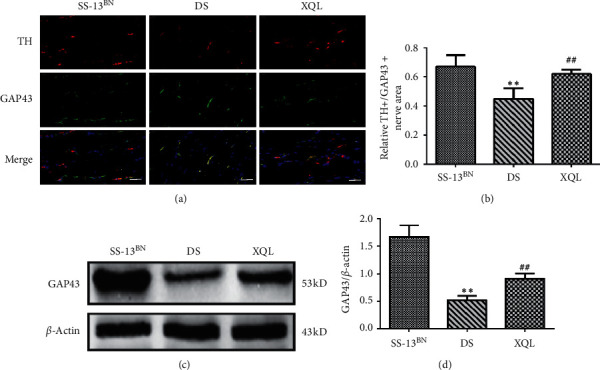
Treatment with XQLT regulated the anatomical dominance of sympathetic nerves. (a) Double immunofluorescence of TH+/GAP43+ positive fibers in left ventricle (scale bars:50 *μ*m). (b) Relative TH+/GAP43+ positive fibers area. (c) Representative Western blot of myocardial GAP43. (d) Relative protein level was calculated by band intensity against *β*-actin. Quantitative data are shown as the mean ± SD, *n* = 9. ^*∗∗*^*P* < 0.01 vs. the SS-13^BN^ group; ^##^*P* < 0.01 vs. the DS group.

GAP43 is a marker of neuronal growth cones which is associated with the nerve ending development [23]. The double immunofluorescence results showed that compared to the SS-13BN group, the expressions of TH+/GAP43+ positive fibers were decreased in DS and XQL groups (*P* < 0.01), while the TH + expressions were significantly decreased. The coexpression of the GAP43+ positive fiber region was observed in some TH + nerves. Postintervention, XQLT significantly increased TH+ and GAP43+ positive fibers, compared to those of the DS group (*P* < 0.01, Figures 4(a) and 4(b)). Similarly, Western blot analysis showed that compared to the SS-13BN group, GAP43 protein levels of DS and XQL groups were decreased (*P* < 0.01). XQLT significantly increased GAP43 compared to that of the DS group (*P* < 0.01, Figures 4(c) and 4(d)).

### 3.5. XQLT Improved the Functional Dominance of Sympathetic Nerves

**Figure 5:**
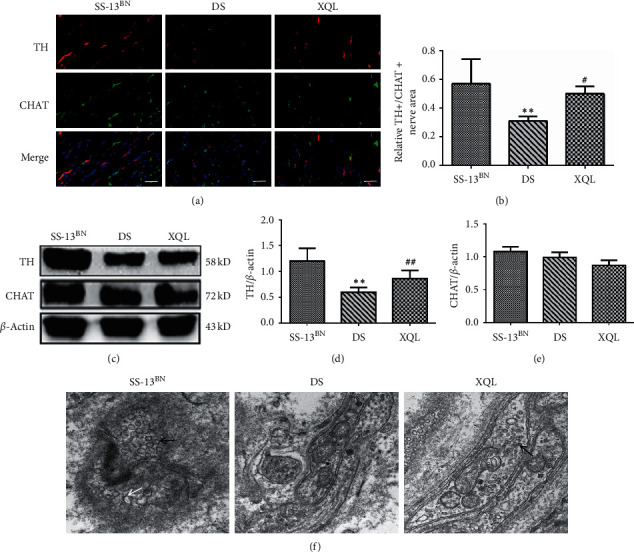
XQLT improved the functional dominance of sympathetic nerves. (a) Double immunofluorescence of TH+/CHAT + positive fibers in the left ventricle (scale bars: 50 *μ*m). (b) Relative TH+/CHAT + positive fibers area. (c) Representative Western blot of myocardial TH and CHAT. (d) and (e) Relative protein levels of TH and CHAT were calculated by band intensity against *β*-actin. (f) The SGVs and SAGVs of left ventricles by transmission electron microscopy (black arrows indicate the SGV and white arrows indicate the SAGV, scale bars: 500 nm). Quantitative data are shown as the mean ± SD, *n* = 9. ^*∗∗*^*P* < 0.01, ^##^*P* < 0.01 vs. the SS-13^BN^ group; ^#^*P* < 0.05 vs. the DS group.

TH reflects sympathetic activity, and CHAT is a parasympathetic marker [24, 25]. According to the double immunofluorescence analysis, compared to the SS-13BN group, the expressions of TH+/CHAT + positive fibers were decreased in the DS and XQL groups due to decreased TH + positive nerves (*P* < 0.01). The level of TH+/CHAT + positive nerve fibers in the XQL group was significantly higher than that in the group (*P* < 0.05, Figures 5(a) and 5(b)). Furthermore, Western blot analysis showed that compared to the SS-13BN group, TH protein expressions in DS and XQL groups decreased (*P* < 0.01), but CHAT expressions had no significance. Compared to the DS group, XQLT significantly increased TH protein expression (*P* < 0.01), and CHAT expression was relatively downregulated, but there was no statistical difference (Figures 5(c), 5(d), and 5(e)).

Next, TEM was used to observe the functional changes from catecholaminergic to cholinergic activity in the sympathetic nerves. The results showed that many SGVs were observed in the SS-13BN group that were intact in shape and uniform in texture, whereas the sympathetic nerve endings in the DS group had both SGVs and a large amount of SAGVs accompanied by vesicle deformation and severe vesicle marginal damage. After XQLT treatment, compared to the DS group, XQLT reduced ultrastructural damage in the left ventricular sympathetic nerve ending (Figure 5(f)).

## 4. Discussion

In recent years, modern treatment strategies for HFrEF which are based on traditional Western medicine, such as angiotensin-converting enzyme inhibitors (ACEI), *β*-blockers, and aldosterone antagonists, are combined with inhibition of neuroendocrine sympathetic nerves and improvement of myocardial remodeling. However, overall, HFrEF remains a major health problem worldwide. Herein, we report that XQLT has a therapeutic potential aimed at regulating the imbalance of cardiac sympathetic innervation for HFrEF.

HF caused by salt-sensitive hypertension is regarded to be very close in pathophysiology to the process of human HF [26]. Other HF models, such as coronary artery ligation or thoracic aortic constriction-induced HF models, could cause sympathetic nerve damage around the arteries, which causes errors in the measurement of various parameters of the sympathetic nervous system during the experiment. Doxorubicin-induced HF causes damage to the autonomic nerves. Therefore, we used DS rats to induce the HF model.

Sympathetic nervous system remodeling is an important pathological mechanism in the development of HF. Several studies show that, in terms of function, the sympathetic nerve is in a state of compensatory activation in the early stage of HF development. As the disease progresses, sympathetic overactivation causes ventricular remodeling [2, 3]. In the process of turning to HFrEF, there are obstacles in the synthesis, release, and reabsorption of NA at the end of the cardiac sympathetic nerve, which leads to the decrease of the sympathetic nerve function persistence. On the other hand, the dominant density of the cardiac sympathetic nerve first increased, then decreased, and finally disappeared [27].

As the neurotransmitter of sympathetic nerves, NA reflects sympathetic nervous system activity [28]. The synthesis and release of NA also affect the myocardium. In HFrEF, the synthesis and reuptake of NA at the nerve endings are significantly reduced, and the level of myocardial NA is decreased. In addition, the density of adrenergic receptors and the sensitivity of myocardium to NA is decreased; therefore, myocardial damage gets worse [29]. The increase in the plasma NA level is caused by the increased sodium overflow rate of myocardial cells, which entails certain toxicity to the myocardium, causes myocardial cell apoptosis, and reduces the functional metabolism of myocardium. The mechanism of intracellular NA overflow and concentration reduction is not clear, but some studies have shown that the expression and activity of NA transporters in neurons are related to the increased NA release [30]. Consistent with these findings, we found that myocardial NA was decreased, while plasma NA was increased in HFrEF rats, suggesting that cardiac sympathetic nerve activity was impaired. This was significantly ameliorated by XQLT which reduced myocardial NA overflow, improved sympathetic nerve damage, and restored some sympathetic nerve functions. As expected, the results revealed that the treatment of XQLT contributed to the beneficial effects on the progression of HFrEF, such as the reductions in weight loss and blood pressure and improvement of heart function, which were associated with the recovery of cardiac sympathetic nerves.

After myocardial injury, Waller's degeneration occurs in cardiac nerve fibers, which may lead to neuronal cell proliferation and axonal regeneration. This regeneration process is triggered by the upregulation of growth factors in nonneuronal cells surrounding the injury site, particularly NGF. NGF has the ability to regenerate cardiac nerves after injury [31] as well as promote cardiomyocyte proliferation, sympathetic differentiation, sympathetic survival, and synaptic activity. It has been reported that the expression of NGF in the myocardium corresponds to the level of sympathetic innervation density [32]. Previous studies have shown that stress overload through the endogenous ET-1/NGF pathway leads to excessive innervation in the ventricle [8, 33]. NGF may stimulate axonal growth through the action of the p75 neurotrophin receptor and the TrkA receptor in sympathetic neurons [34–37]. GAP43 is a marker of neuronal growth cones, associated with the quantification of nerve fiber density [8, 23]. Therefore, the expression of GAP43 is used to indicate the extent to which NGF affects cardiac sympathetic nerve regeneration. Our data suggested that the expressions of NGF and GAP43 in rats with HF were lower than that in the SS-13BN group, and the immunofluorescence also showed a reduction in the density of sympathetic nerves. A high level of plasma NA reduces NGF in the myocardium, and this has been reported in previous studies [9]. Our data also indicated that the increased plasma NA in HFrEF rats was accompanied by decreased NGF. After XQLT intervention, the expression of NGF and GAP43 in HFrEF rats increased, and the intracellular NA overflow decreased, suggesting the regeneration of cardiac sympathetic nerves and increased anatomical innervation. The results showed that the beneficial effects of XQLT on the HFrEF rats may be associated with improving the level of NGF and promoting the regeneration of cardiac sympathetic nerve fibers.

However, anatomical imbalance of dominance is accompanied by the loss of sympathetic nerve function, which is manifested by the conversion of sympathetic nerves to cholinergic nerves. This process is induced by the gp130 signal in the heart muscle-derived cholinergic differentiation factor [19], including members of LIF and interleukin-6 (IL-6) family [38], which are specifically expressed as the sympathetic NA synthesis rate-limiting enzyme TH (sympathetic marker) decreases and the acetylcholine synthesis rate-limiting enzyme CHAT (parasympathetic markers) increases. Our study found that in rats with HF, the level of LIF was increased, TH was downregulated, and SGVs involving catecholamine of cardiac sympathetic nerve endings were reduced, and SAGVs involving Ach were observed under TEM. In the XQL group, LIF was decreased, the expression of TH+/CHAT + positive fibers was increased, and the microstructure of sympathetic nerve endings was improved. These results indicated that elevated LIF promoted cholinergic transdifferentiation and sympathetic dysfunction in HF. Treatment with XQLT reduced the expression of LIF, inhibited the conversion of sympathetic nerves to parasympathetic nerves, and regulated the functional dominance of sympathetic nerves in HFrEF.

In general, these results demonstrated that cardiac sympathetic nerve density and function were decreased in rats with HFrEF, and XQLT regulated the imbalance of sympathetic nerve anatomy and function by increasing NGF and reducing LIF, which may be a new direction for HFrEF treatment in the future. Due to multicomponent and multitarget effects, this traditional Chinese medicine demonstrated great potential in the clinical treatment of HFrEF.

## 5. Conclusion

XQLT, as a traditional Chinese prescription, maintains cardiac function during HFrEF in salt-sensitive rats by regulating the anatomical/functional dominance imbalance of the sympathetic nerve. These findings suggested that XQLT could be an effective therapeutic strategy for the treatment of HFrEF.

## Data Availability

The datasets used and/or analyzed during the current study are available from the corresponding author upon request.
